# Screening and Treating UN Peacekeepers to Prevent the Introduction of Artemisinin-Resistant Malaria into Africa

**DOI:** 10.1371/journal.pmed.1001822

**Published:** 2015-05-05

**Authors:** Stan Houston, Adam Houston

**Affiliations:** 1 Department of Medicine and School of Public Health, University of Alberta, Edmonton, Alberta, Canada; 2 Institute for Justice & Democracy in Haiti, Boston, Massachusetts, United States of America

## Abstract

Stan Houston and Adam Houston highlight how deployment of UN peacekeepers has the potential to introduce artemisinin-resistant malaria into Africa.

**Summary Points**

- The Haitian cholera epidemic provides a tragic demonstration of the potential for United Nations peacekeepers to introduce serious disease into vulnerable populations.
- Resistance to artemisinin derivatives, now the global standard therapy for falciparum malaria, has emerged and is spreading in Southeast Asia.
- UN peacekeeping troops from Southeast Asia are frequently deployed in sub-Saharan Africa.
- These circumstances entail a high risk of introducing artemisinin resistance into the populations most affected by malaria, with potentially disastrous consequences for malaria treatment and control in sub-Saharan Africa.
- The UN has a responsibility to prevent such an outcome; selective predeployment screening and treatment of UN peacekeeping troops is feasible and urgently needed.

## Introduction: The Precedent of Cholera in Haiti

In the aftermath of the massive earthquake that devastated Haiti in 2010, an ongoing epidemic of cholera introduced by United Nations peacekeepers has resulted in over 730,000 cases and over 8,700 deaths—the largest single-country cholera epidemic in nearly a century [1,2]. This disaster should serve as an urgent warning about the potential for introduction by UN troops of other serious infectious diseases into the vulnerable populations they were sent to protect. Indeed, the UN has recently agreed to avoid rotation of African troops to Haiti because of concern about the introduction of Ebola [3]. But the tragedy in Haiti pales in comparison to the scale of long-term impact on malaria morbidity, mortality, and control programs that would result from the introduction of artemisinin-resistance into sub-Saharan Africa, where 85% of the world’s falciparum malaria cases and over 90% of all malaria deaths now occur [4]. This threat demands urgent action, in particular on the part of the UN.

## The Importance of Artemisinins and the Threat of Resistance

Artemisinin derivatives are currently the mainstay of antimalarial treatment throughout the world. Their implementation, along with expanded use of insecticide-treated bed nets, accounts for a large part of the reduction in malaria deaths in Africa over the past decade [5]. Consequently, the emergence of decreasing responsiveness to artemisinin derivatives over the past few years is deeply concerning. Initially observed in Cambodia, resistant strains now appear to be spreading rapidly within the region and have been observed in Myanmar, Laos, Thailand, Vietnam, and, most recently, at the Indian border [6–8]. This pattern of rapid dissemination evokes the history of chloroquine resistance, which first appeared in the same area of Southeast Asia over 50 years ago. Chloroquine resistance soon reached Africa, where its inexorable spread across the continent over a few years resulted in the loss of a safe, inexpensive treatment and a 2-to-3-fold increase in malaria deaths and admissions for severe malaria [9,10]. With no comparably effective treatment alternative available, the establishment of artemisinin-resistant malaria in sub-Saharan Africa would be expected to result in a substantial reversal of the recent progress in malaria control and a major increase in malaria illness and death.

New WHO initiatives to prevent the emergence of artemisinin resistance by ensuring the drug is only available coformulated with other antimalarials represent an important positive step, to the degree that they are effectively implemented. Any positive impact of these policies, however, would be rapidly overwhelmed if resistant parasites were introduced directly into the fertile soil of a highly malaria-endemic population.

## Peacekeepers and Malaria Transmission

UN peacekeeping entails a particularly potent mix of risk factors favoring the spread of artemisinin resistance. Many countries where peacekeepers are deployed are also among the most vulnerable to malaria, including South Sudan, the Democratic Republic of the Congo, and the Central African Republic. This vulnerability is exacerbated by mass population displacements and damage to health systems as a result of conflict. Peacekeepers working in such conditions are at risk of exposure to local mosquito vectors, making them both potential victims of malaria and contributors to its transmission. In at least one nonmalarious country, returning UN peacekeepers are the largest single risk group for imported malaria [9]. At the same time, peacekeeping forces increasingly come from malaria-endemic countries, including those where artemisinin resistance has been documented. Among current UN missions, Cambodia has contributed troops to those in Mali, South Sudan, and the Darfur and Abyei regions, while Thailand has participated in Darfur, Cote d’Ivoire, and the Central African Republic. Vietnam recently began contributing troops in South Sudan [11].

Existing UN guidelines for peacekeepers focus on protecting them from local infectious diseases, ignoring the potential for diseases imported by the peacekeepers to pose a threat to locals, as was all too readily illustrated in Haiti. The potential for peacekeepers to import drug-resistant malaria into a previously drug-sensitive region has already been demonstrated: following a tour of duty in the Democratic Republic of the Congo, UN peacekeepers brought chloroquine-resistant malaria home to Guatemala [12]. Ultimately, it would take only one soldier carrying resistant parasites to introduce resistance into an entire continent under the much more favorable conditions for ongoing transmission that prevail in Africa [13].

## A Feasible Preventive Intervention

Fortunately, robust, affordable technology is readily available that would enable routine screening of troops from endemic areas prior to deployment, in a manner both more feasible and more reliable than screening for many other diseases such as cholera. Soldiers from malaria-endemic areas, particularly those where, based on current surveillance, artemisinin resistance is present or suspected, could be screened using polymerase chain reaction (PCR) methodology. Any individual found positive for *Plasmodium falciparum*, regardless of symptoms, would have his or her deployment delayed until he or she was cured. The specific treatment used would be chosen on the basis of the best current evidence. It would be followed by primaquine treatment as per WHO recommendations, with the need for prior glucose-6-phosphate dehydrogenase (G6PD) testing determined by the dosing strategy used [14], to eradicate gametocytes of the potentially resistant malaria strain. Soldiers would continue to take the UN-recommended prophylaxis and other preventive measures during their deployment.

The sensitivity of PCR for malaria is much greater than that of microscopy or rapid diagnostic tests and continues to improve [15,16]. Since asymptomatic, semi-immune individuals with low parasitemias may transmit malaria [17], it is critical that the most sensitive available technology be used. The proposed strategy need not initially depend on identifying artemisinin resistance genes, an evolving technology [18,19].

This predeployment screening would also benefit the individual peacekeepers; those found to be positive would gain from the diagnosis and cure of an infection that might otherwise have recrudesced in the field.

Although the threat posed by artemisinin resistance is sufficiently great that a case could be made for broader application of this screen-and-treat strategy to other travelers, UN soldiers constitute both the highest priority and the most feasible target group for several reasons: (1) the interaction of these troops with local populations is likely to be uniquely high risk in regard to malaria transmission, (2) UN missions exist under a single centralized authority, and their military structure makes implementation and enforcement relatively straightforward, and (3) the UN has an ethical obligation to not cause harm through peacekeeping activities.

While we believe the approach described here represents the best option given current knowledge, we emphasize that the priority is effective action on this issue. Because of concerns regarding the sensitivity of currently available tests in detecting all malaria infections, other approaches have been suggested, such as treatment (including primaquine) before deployment and without malaria testing of all soldiers from endemic countries, even though only a small minority will have malaria. Another element of the response to the threat of spreading artemisinin resistance, particularly in Africa, should be the implementation of systematic surveillance for resistance, which will be facilitated by the development and validation of molecular markers [13,19].

While it is simpler to screen for malaria than for cholera, it is much more difficult to contain malaria once introduced. Compliance with basic principles of sanitation would have kept *Vibrio cholerae*–infected human waste out of Haitian waterways and cholera from the Haitian population. By contrast, numerous studies indicate the difficulties of protecting troops from malaria, indicating that it is nearly impossible to reliably prevent peacekeeping forces from exposure that could lead to acquisition or transmission of the infection. Today, the cost of eradicating cholera from the island of Hispaniola is estimated at US$2.2 billion [20]. Eradicating drug-resistant malaria from areas where eliminating the disease has historically proven impossible is not likely to be achievable, much less affordable. Consequently, it is crucial to prevent introduction of drug resistance in the first place.

## Urgent Action Is Indicated, for Which the UN Has a Primary Responsibility

Cambodia's National Malaria Center has recognized the risk posed by its peacekeepers, and it appears the Cambodian military has taken steps towards developing and implementing a screening framework [21], a commendable initiative. However, artemisinin resistance poses a global threat that should properly be addressed by the international body responsible for peacekeepers. Both ethically and logistically, the development and implementation of standards for screening and treatment should be the responsibility of the UN as part of a comprehensive commitment to disease prevention among both peacekeepers and the local populations they serve. These measures can and should be implemented immediately.

## References

[1] Pan American Health Organization. Epidemiological Update Cholera, 27 June 2014. Reliefweb. 27 June 2014. http://reliefweb.int/report/haiti/epidemiological-update-cholera-27-june-2014.

[2] Gaudart J, Rebaudet S, Barrais R, Boncy J, Faucher B, Piarroux M, et al Spatio-temporal dynamics of cholera during the first year of the epidemic in Haiti. PLoS Negl Trop Dis. 2013;7: e2145 10.1371/journal.pntd.0002145 23593516 PMC3617102

[3] Belt R. Haiti: UN troops rotation from Africa suspended to keep Ebola away. Haitian-Caribbean News Network. 16 October 2014. http://hcnn.ht/en/2014_10/politics/406/Haiti-UN-troops-rotation-from-Africa-suspended-to-keep-Ebola-away.htm.

[4] World Health Organization. 10 Facts on Malaria in Africa. http://www.afro.who.int/en/clusters-a-programmes/dpc/malaria/features/2287-10-facts-on-malaria-in-africa.html.

[5] White NJ, Pukrittayakamee S, Hien TT, Faiz MA, Mokuolu OA, Dondorp AM. Malaria. Lancet. 2014;383: 723–735. 10.1016/S0140-6736(13)60024-0 23953767

[6] World Health Organization. Status report on artemisinin resistance. January 2014. http://www.who.int/malaria/publications/atoz/status_rep_artemisinin_resistance_jan2014.pdf.

[7] Ashley EA, Dhorda M, Fairhurst RM, Amaratunga C, Lim P, Suon S, et al Spread of artemisinin resistance in Plasmodium falciparum malaria. N Engl J Med. 2014;371: 411–423. 10.1056/NEJMoa1314981 25075834 PMC4143591

[8] Tun KM, Imwong M, Lwin KM, Win AA, Hlaing TM, Hlaing T, et al Spread of artemisinin-resistant Plasmodium falciparum in Myanmar: a cross-sectional survey of the K13 molecular marker. Lancet Infect Dis 15: 415–421. 10.1016/S1473-3099(15)70032-0 25704894 PMC4374103

[9] Meneizel S, Rabadi K, Muhareb H, Kawar G. Epidemiology of imported malaria cases in Jordan between 2000 and 2005. JRMS. 2009;16: 10–15.

[10] Trape JF, Pison G, Preziosi MP, Enel C, Desgrees du Lou A, Delaunay V, et al Impact of chloroquine resistance on malaria mortality. C R Acad Sci III. 1998;321: 689–697. 9769862 10.1016/s0764-4469(98)80009-7

[11] United Nations (2015) Current Peacekeeping Operations. http://www.un.org/en/peacekeeping/operations/current.shtml.

[12] Juliao PC, Sosa S, Gonzalez LD, Padilla N, Ortiz L, Goldman I, et al Importation of chloroquine-resistant Plasmodium falciparum by Guatemalan peacekeepers returning from the Democratic Republic of the Congo. Malar J. 2013;12: 344 10.1186/1475-2875-12-344 24060234 PMC3849196

[13] Talisuna AO, Karema C, Ogutu B, Juma E, Logedi J, Nyandigisi A, et al Mitigating the threat of artemisinin resistance in Africa: improvement of drug-resistance surveillance and response systems. Lancet Infect Dis. 2012;12: 888–896. 10.1016/S1473-3099(12)70241-4 23099083 PMC3555126

[14] WHO Evidence Review Group. The Safety and Effectiveness of Single Dose Primaquine as a P. falciparum gametocytocide. Pullman Hotel, Bangkok, Thailand, 13–15 August 2012. Malaria Policy Advisory Committee Meeting 11–13 September 2012, WHO HQ Session 5. 2012;1–19. http://www.who.int/malaria/mpac/sep2012/primaquine_single_dose_pf_erg_meeting_report_aug2012.pdf.

[15] Imwong M, Hanchana S, Malleret B, Renia L, Day NP, Dondorp A, et al High-throughput ultrasensitive molecular techniques for quantifying low-density malaria parasitemias. J Clin Microbiol. 2014;52: 3303–3309. 10.1128/JCM.01057-14 24989601 PMC4313154

[16] Kamau E, Tolbert LS, Kortepeter L, Pratt M, Nyakoe N, Muringo L, et al Development of a highly sensitive genus-specific quantitative reverse transcriptase real-time PCR assay for detection and quantitation of plasmodium by amplifying RNA and DNA of the 18S rRNA genes. J Clin Microbiol. 2011;49: 2946–2953. 10.1128/JCM.00276-11 21653767 PMC3147742

[17] Bousema T, Okell L, Felger I, Drakeley C. Asymptomatic malaria infections: detectability, transmissibility and public health relevance. Nat Rev Microbiol. 2014;12: 833–840. 10.1038/nrmicro3364 25329408

[18] Ariey F, Witkowski B, Amaratunga C, Beghain J, Langlois AC, Khim N, et al A molecular marker of artemisinin-resistant Plasmodium falciparum malaria. Nature. 2014;505: 50–55. 10.1038/nature12876 24352242 PMC5007947

[19] Miotto O, Amato R, Ashley EA, MacInnis B, Almagro-Garcia J, Amaratunga C, et al Genetic architecture of artemisinin-resistant Plasmodium falciparum. Nat Genet. 2015;47: 226–234. 10.1038/ng.3189 25599401 PMC4545236

[20] Olson A. UN Launches $2.27 Billion Cholera Plan For Haiti, Dominican Republic But Needs Funds. The Huffington Post. 12 November 2012. http://www.huffingtonpost.com/2012/12/11/un-cholera-plan_n_2281279.html.

[21] Hruby D. RCAF in Africa; Fears of Drug Resistant Malaria. The Cambodia Daily. 1 March 2014. http://www.cambodiadaily.com/archives/rcaf-in-africa-fears-of-drug-resistant-malaria-53384/.

